# Systemic Administration of α7-Nicotinic Acetylcholine Receptor Ligands Does Not Improve Renal Injury or Behavior in Mice With Advanced Systemic Lupus Erythematosus

**DOI:** 10.3389/fmed.2021.642960

**Published:** 2021-04-13

**Authors:** Jessica Y. Morales, Cassandra M. Young-Stubbs, Caroline G. Shimoura, William R. Kem, Victor V. Uteshev, Keisa W. Mathis

**Affiliations:** ^1^Department of Physiology and Anatomy, University of North Texas (UNT) Health Science Center, Fort Worth, TX, United States; ^2^Department of Pharmacology and Therapeutics, University of Florida, Gainesville, FL, United States; ^3^Department of Pharmacology and Neuroscience, University of North Texas (UNT) Health Science Center, Fort Worth, TX, United States

**Keywords:** SLE, cholinergic anti-inflammatory pathway, positive allosteric modulators, renal injury, renal inflammation, behavior

## Abstract

There is a critical need for safe treatment options to control inflammation in patients with systemic lupus erythematosus (SLE) since the inflammation contributes to morbidity and mortality in advanced disease. Endogenous neuroimmune mechanisms like the cholinergic anti-inflammatory pathway can be targeted to modulate inflammation, but the ability to manipulate such pathways and reduce inflammation and end organ damage has not been fully explored in SLE. Positive allosteric modulators (PAM) are pharmacological agents that inhibit desensitization of the nicotinic acetylcholine receptor (α7-nAChR), the main anti-inflammatory feature within the cholinergic anti-inflammatory pathway, and may augment α7-dependent cholinergic tone to generate therapeutic benefits in SLE. In the current study, we hypothesize that activating the cholinergic anti-inflammatory pathway at the level of the α7-nAChR with systemic administration of a partial agonist, GTS-21, and a PAM, PNU-120596, would reduce inflammation, eliminating the associated end organ damage in a mouse model of SLE with advanced disease. Further, we hypothesize that systemic α7 ligands will have central effects and improve behavioral deficits in SLE mice. Female control (*NZW*) and SLE mice (*NZBWF1*) were administered GTS-21 or PNU-120596 subcutaneously via minipumps for 2 weeks. We found that the increased plasma dsDNA autoantibodies, splenic and renal inflammation, renal injury and hypertension usually observed in SLE mice with advanced disease at 35 weeks of age were not altered by GTS-21 or PNU-120596. The anxiety-like behavior presented in SLE mice was also not improved by GTS-21 or PNU-120596. Although no significant beneficial effects of α7 ligands were observed in SLE mice at this advanced stage, we predict that targeting this receptor earlier in the pathogenesis of the disease may prove to be efficacious and should be addressed in future studies.

## Introduction

Chronic inflammation is inevitable in systemic lupus erythematosus (SLE) and it contributes to the morbidity and mortality of the disease (1–3). Nearly all organ systems are affected by inflammation during SLE. For example, there is renal injury due to nephritis that can result in hypertension as well as central nervous system inflammation that can result in unfavorable behavioral phenotypes, among others (4, 5). Because chronic inflammation is an underlying mechanism in SLE and its complications, it is a justifiable target to improve outcomes. The current standard of care in SLE patients includes glucocorticoid therapy that provides overall immunosuppression but comes with adverse side effects like weight gain, mood swings, upset stomach, headache, dizziness, insomnia, depression. The use of biologics, an important class of drugs that usually target one cytokine, has not been effective in SLE clinical trials possibly because multiple immune cells and cytokines are involved in the disease process. Therefore, there is a critical need for safe alternative treatment options to broadly control inflammation in patients with SLE.

The cholinergic anti-inflammatory pathway is a neuroimmune mechanism by which the autonomic nervous system controls inflammatory processes (6–9). The cholinergic anti-inflammatory pathway has been shown to be relevant in many disease models, including autoimmune diseases (10–17). In this pathway, stimulation of the vagus nerve increases splenic nerve activity and ultimately inhibits the release of pro-inflammatory cytokines from splenic macrophages and other immune cells. The latter requires activation of the α7 subunit-containing nicotinic acetylcholine receptor (α7-nAChR) expressed in these splenic immune cells. It is well-accepted that the α7-nAChR is a critical component of this nerve-to-spleen pathway (15); the lack of α7-nAChRs exacerbates inflammatory diseases (15, 18–21). There are no studies that dispute the protective role of α7-nAChR stimulation in reducing inflammation: in fact, the benefits of α7-nAChR stimulation are explained in studies of the classic pathway described by Kevin Tracey as well as the alternative tissue-specific pathways described by others (22–25). For these reasons, drugs that can augment activation of α7-nAChRs may prove to be a viable therapeutic option for many diseases associated with chronic inflammation, and especially SLE. Indeed, our preliminary and published studies support this hypothesis by demonstrating that sub-chronic administration (7 days) of nicotine, a non-selective α7-nAChR agonist, protected kidneys by significantly reducing renal inflammation and renal injury in a mouse model of SLE (26). However, nicotine cannot be used as a therapy in humans because of its addictive and toxic nature. Thus, a solid proof-of-principle study is required to demonstrate that non-addictive, non-toxic cholinergic agents can be as effective as nicotine in producing significant anti-inflammatory efficacy in SLE but without severe side effects. Furthermore, as these recent results are promising so it would benefit the field to determine if targeting the α7-nAChR by global means can also be beneficial in other organs like the central nervous system and alter behavior in SLE.

Positive allosteric modulators (PAM) and selective partial agonists of α7-nAChRs can be used to augment endogenous α7-mediated cholinergic tone and thus, augment α7-dependent anti-inflammatory activity. In this study, we used GTS-21, a partial agonist, and PNU-120596 (i.e., PNU), a prototypical type-II α7 PAM, to test their effects on chronic inflammation in a mouse model of SLE. The protective anti-inflammatory efficacy of these classes of α7 ligands has been established in various animal models of stroke and traumatic brain injury (27–37). Since α7-nAChRs are essential in the anti-inflammatory response within the cholinergic anti-inflammatory pathway, we hypothesize that systemic administration of α7 ligands will be a successful therapeutic strategy in SLE by reducing peripheral inflammation and improving behavior in a mouse model of SLE. Because of the ubiquitous expression of α7-nAChRs in neuronal, glial and immune tissues, selective α7 agents are uniquely positioned as promising neuroprotective, anti-inflammatory and pro-cognitive combinational treatments.

## Materials and Methods

### Experimental Design

Female and SLE (*NZBWF1*) and control (*NZW/LacJ*) mice were obtained from Jackson Laboratories (Bar Harbor, ME) at 4–6 weeks of age and aged in a temperature-controlled facility with free access to food and water. Control and SLE mice were randomly assigned to 3 groups each: control/saline, control/GTS, control/PNU, SLE/saline, SLE/ GTS, and SLE/PNU. At 33 weeks of age, drugs were administered subcutaneously via Alzet mini-pumps for 2 weeks. Blood samples were taken (retroorbitally, ~100 μL) and the beginning and end of the study in order to measure anti-dsDNA autoantibodies associated with SLE via ELISA and albuminuria was obtained weekly following 24 h metabolic cage collection of urine. Both anti-dsDNA autoantibodies and albuminuria are important determinants of disease severity in disease. Body weight was monitored throughout the study and at the end of experiments, animals were euthanized and tissues harvested and stored for later biochemical analysis. All animal studies utilized ARRIVE guidelines (38) and were approved by the University of North Texas Health Science Center's Institutional Animal Care and Use Committee (IACUC) in accordance with National Institutes of Health (NIH) Guide for the Care and Use of Laboratory Animals.

### Drugs

GTS-21 was made in house at the University of Florida as described previously (39). PNU-120596 was purchased from Selleckchem (Houston, TX). Drugs were dissolved in dimethyl sulfoxide (DMSO; vehicle) to create an 80 mM stock solution that was kept frozen at −30°C until the day of experiments. The amount of DMSO injected in each animal did not exceed 0.5 ml/kg. Analytical data for GTS-21 including melting point have been reported previously (39). Purity was ≥98% as determined by proton NMR analysis. In the current study, GTS-21 was administered subcutaneously at a dose of 15 mg/kg/day and PNU-120596 was administered subcutaneously at a dose of 10 mg/kg/day. Drugs administered at 4–20 mg/kg/day were found effective in other studies (37, 40–42).

### dsDNA Autoantibody Detection

Anti-double-stranded DNA (dsDNA) autoantibodies were measured in plasma samples acquired at the end of the study via ELISA (Alpha Diagnostic International, San Antonio, TX, USA) per the manufacturer's instructions as previously described (4, 43–46).

### Assessment of Peripheral Inflammation

Splenic as well as renal cortical and medullary TNF-α expression were assessed via Western blot and normalized to total protein as previously described (46).

### Indices of Renal Injury and Blood Pressure Measurements

Urine was collected throughout the study, which allowed assessment of urinary albumin by ELISA and calculation of albumin excretion rate as previously reported (26, 46, 47).

At 35 weeks of age, a subset of anesthetized mice was implanted with carotid artery catheters in order to measure mean arterial pressure before euthanization as previously described (43–47). Blood pressure was collected in conscious mice for 1.5 h between the hours of 8 am and 12 noon for 2 consecutive days.

### Behavioral Assessment

Standard assays for sensorimotor and emotional deficits were conducted in a subset of mice pre- (day 0) and post- (day 15) treatment to assess behavioral effects of GTS-21 and PNU in SLE mice. Each mouse was tested at both time points to minimize the number of animals used and correlate performance in various assays. The open field (OF) test was used to evaluate sensorimotor deficits and anxiety. Anxiety was additionally evaluated in the light-dark box (LDB) test. Depression was evaluated in the tail suspension (TS) test. The following order of tests was followed in each experimental day: ≥0.5 h accommodation→OF→≥1 h rest→LDB→ ≥1 h rest→TS. In OF experiments, each mouse was placed for 11 min in an opaque chamber (40.5 × 40.5 × 30.5 cm) made of polyvinyl chloride. The spontaneous locomotion within the chamber was recorded by a video camera mounted above the chamber and analyzed by All-Maze Video Tracking Software (Orchid Scientific Ltd, Maharashtra, India). Forward locomotion in centimeters, sleep time and time spent in the chamber center were determined. After OF tests, the animals were given at least 1 h rest before subjecting them to LDB tests. In LDB experiments, each mouse was placed for 10 min in an opaque acrylic chamber (43 × 29 × 20 cm). One half of the chamber was exposed to direct light from above, while the other half was protected from light by an opaque carton lid. The amount of time the mouse spent in each chamber was recorded by a video camera mounted above the chamber. In TS experiments, each mouse was suspended in the air for 6 min by its tail taped to a plastic cord attached to a horizontal bar mounted to a stand to ensure that 10–15 cm distance separates the mouse from the surface underneath. The animal's behavior was recorded on a video camera and analyzed offline. The total immobility time was measured. Behavioral tests were initiated between the hours of 7 and 8 am, but could last throughout the daylight hours. All behavioral protocols have been approved by the IACUC at UNTHSC.

### Statistical Analysis

Statistical analysis was achieved using SigmaPlot 11.0 (Systat, Richmond, CA, USA). Data are calculated as mean ± standard error of the mean. *T*-test or two-way ANOVA, with or without repeated measures followed by the appropriate *post-hoc* test, was used to determine differences between groups, with a *p* < 0.05 indicating a significant difference. The statistical test used for each data set is included within each figure legend.

## Results

Body weight at the conclusion of the study was higher in SLE mice compared to controls, and SLE mice also exhibited splenomegaly (Table 1). GTS-21 and PNU had no effect on body weight or spleen weight. There were no differences between any of the groups in kidney weight or heart weight (Table 1; both normalized to body weight).

**Table 1:** Body and tissue weights (in grams) for control and SLE mice treated with either vehicle, GTS-21, or PNU-120596.

	**Control/Vehicle**	**Control/PNU**	**Control/GTS**	**SLE/Vehicle**	**SLE/GTS**	**SLE/PNU**
Body Weight (BW)	33.1097 ± 1.3219	34.3262 ± 1.2639	33.2249 ± 1.6489	41.9572 ± 2.0512	42.3558 ± 1.6936	43.7956 ± 1.9736
Average Kidney Weight/BW	0.0069 ± 0.0003	0.0065 ± 0.0002	0.0064 ± 0.0002	0.0063 ± 0.0006	0.0060 ± 0.0003	0.0061 ± 0.0004
Spleen Weight	0.1362 ± 0.0081	0.1456 ± 0.0090	0.1264 ± 0.0067	0.17215 ± 0.0244	0.1906 ± 0.0191	0.2165 ± 0.0243
Heart Weight/BW	0.0056 ± 0.0002	0.0056 ± 0.0003	0.0051 ± 0.0003	0.0050 ± 0.0005	0.0050 ± 0.0003	0.0050 ± 0.0003

*Neither GTS-21 nor PNU-120596 had a significant effect on these parameters in control or SLE mice (n = 14–20/per group). Data were analyzed using a two-way ANOVA. [ANOVA results—Body weight: Group (p < 0.001); Treatment (p = 0.782); Interaction (p = 0.712). Average kidney weight: Group (p = 0.209); Treatment (p =0.631); Interaction (p = 0.834). Spleen weight: Group (p < 0.001); Treatment (p = 0.633); Interaction (p = 0.234). Heart weight: Group (p = 0.217); Treatment (p = 0.516); Interaction (p = 0.650)]*.

**Figure 1:**
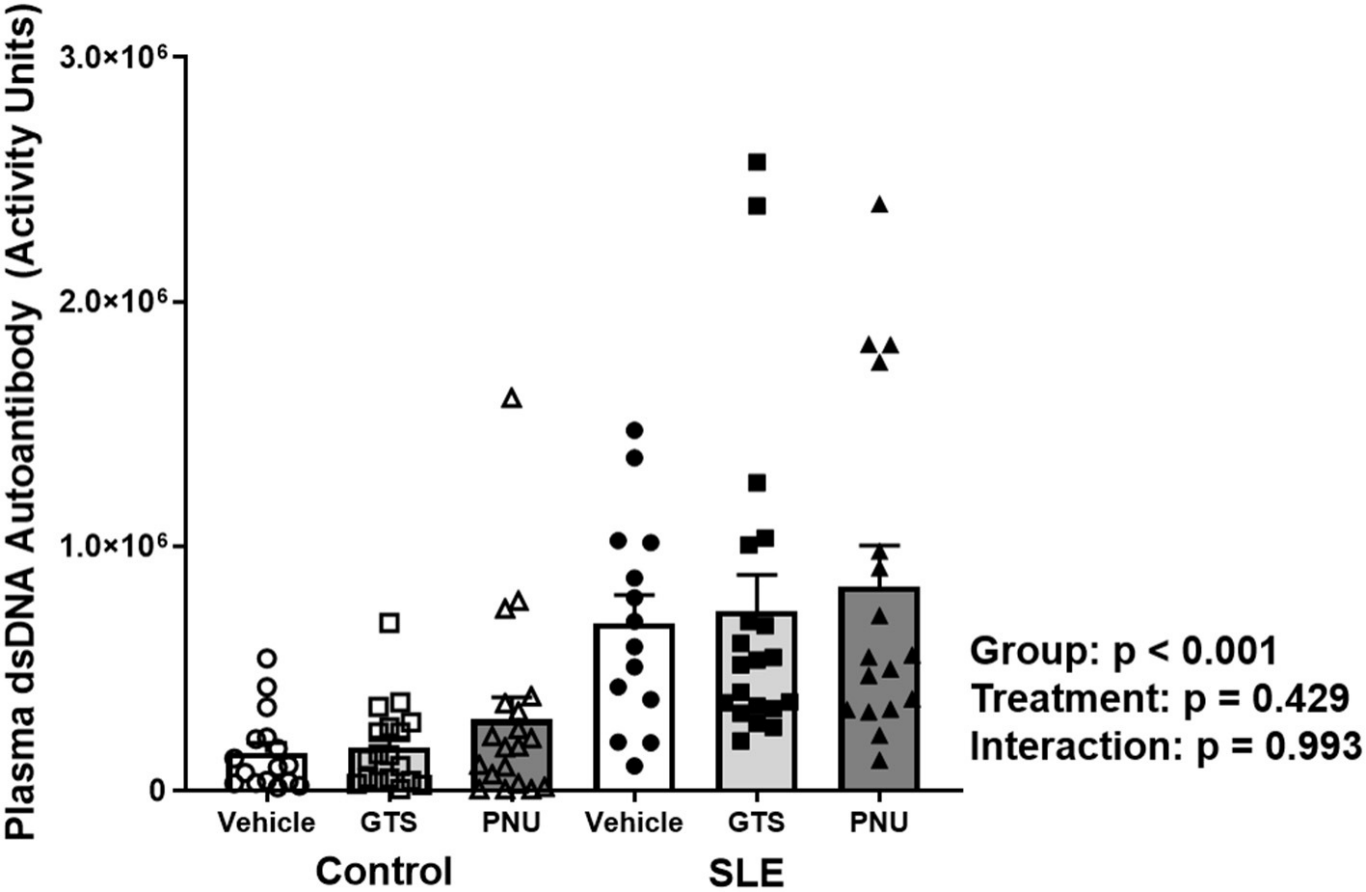
Plasma levels of dsDNA autoantibodies (activity units) in control and SLE mice treated with either vehicle, GTS-21, or PNU-120596. Neither GTS-21 nor PNU-120596 had a significant effect on albumin excretion rate in control or SLE mice (*n* = 14–20 per group). Data were analyzed using a two-way ANOVA and the results of this analysis are listed on the graph.

Disease severity, as indicated by plasma dsDNA autoantibodies (activity units), was higher in SLE mice compared to controls (Figure 1; *p* < 0.001). There was no significant effect of treatment (*p* = 0.429) and there was no interaction (*p* = 0.993) to allow the determination of differences within groups.

**Figure 2:**
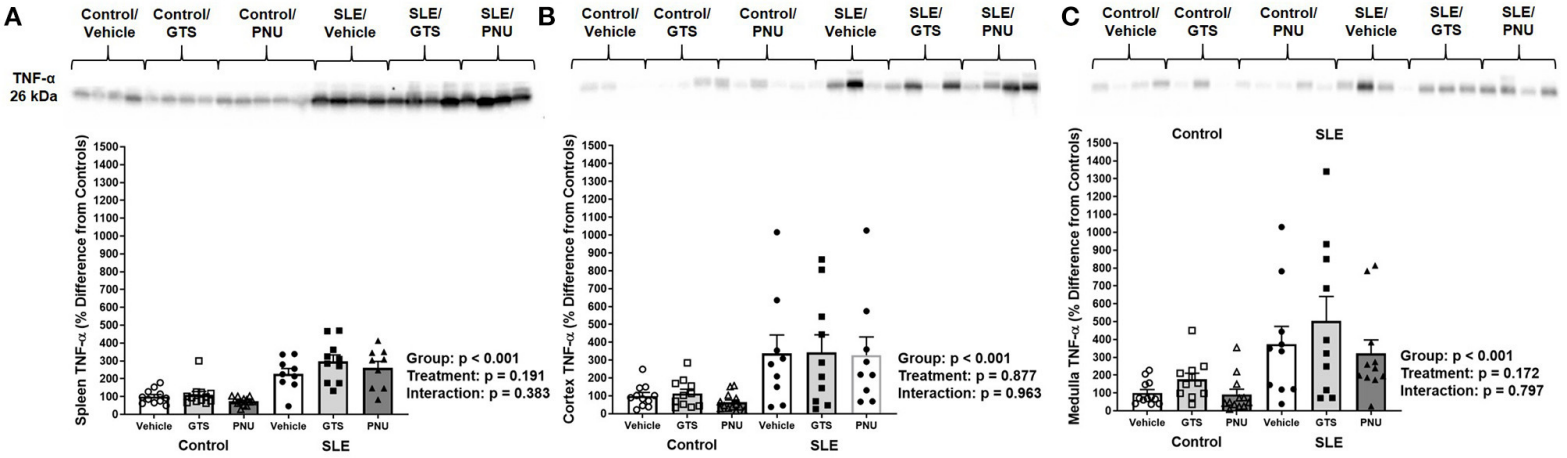
Splenic **(A)**, renal cortical **(B)**, and medullary **(C)** TNF-α (presented as percent difference from controls) in control and SLE mice treated with either vehicle, GTS-21, or PNU-120596. Neither GTS-21 nor PNU-120596 had a significant effect on renal or splenic inflammation in control or SLE mice (*n* = 9–13 per group). Data were analyzed using a two-way ANOVA and the results of this analysis are listed on the graph. Representative blots are included above each figure. The molecular weight of TNF-α is 26 kDa.

To test whether PNU and GTS-21 treatments had any effect on inflammation via the α7-nAChR component of the cholinergic anti-inflammatory pathway, we measured TNF-α in the spleen and kidneys. Splenic, renal cortical, and renal medullary TNF-α were higher in SLE mice compared to controls (Figures 2A–C; all *p* < 0.001). There was no significant effect of treatment (cortical, *p* = 0.877; medullary, *p* = 0.172; splenic, *p* = 0.191) for either set of data and there were no interactions to allow determination of differences within groups.

**Figure 3:**
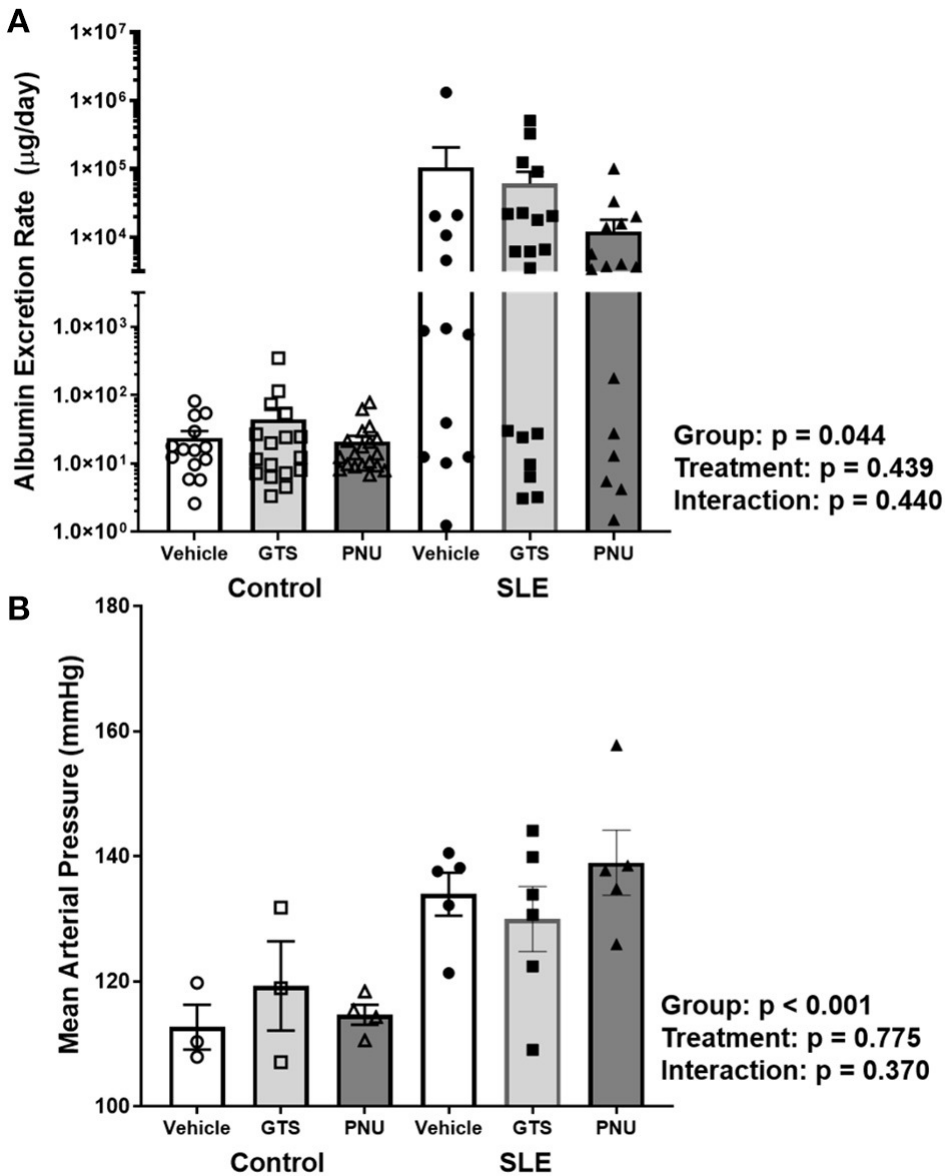
**(A)** Albumin excretion rate (mg/day) in control and SLE mice treated with either vehicle, GTS-21, or PNU-120596. Neither GTS-21 nor PNU-120596 had a significant effect on albumin excretion rate in control or SLE mice (*n* = 13–19 per/group) Data were analyzed using a two-way ANOVA and the results of this analysis are listed on the graph. Note: the y-axis is presented as a log scale. **(B)** Mean arterial pressure (mmHg) in control and SLE mice treated with either vehicle, GTS-21, or PNU-120596. Neither GTS-21 nor PNU-120596 had a significant effect on mean arterial pressure in conscious and freely moving control or SLE mice (*n* = 3–6 per group). Data were analyzed using a two-way ANOVA and the results of this analysis are listed on the graph.

Chronic inflammation contributes to end organ damage during SLE, so we determined the effect of PNU and GTS-21 on the renal injury and hypertension that develops in SLE. Albumin excretion rate, an index of renal injury, was higher in SLE mice compared to controls (Figure 3A; *p* = 0.044). There was no significant effect of treatment (*p* = 0.439) and there was no interaction (*p* = 0.440) to allow the determination of differences within groups. SLE mice were hypertensive with a higher mean arterial pressure than controls (Figure 3B; *p* < 0.001). There was no significant effect of treatment (*p* = 0.775) and there was no interaction (*p* = 0.370) to allow the determination of differences within groups.

**Figure 4:**
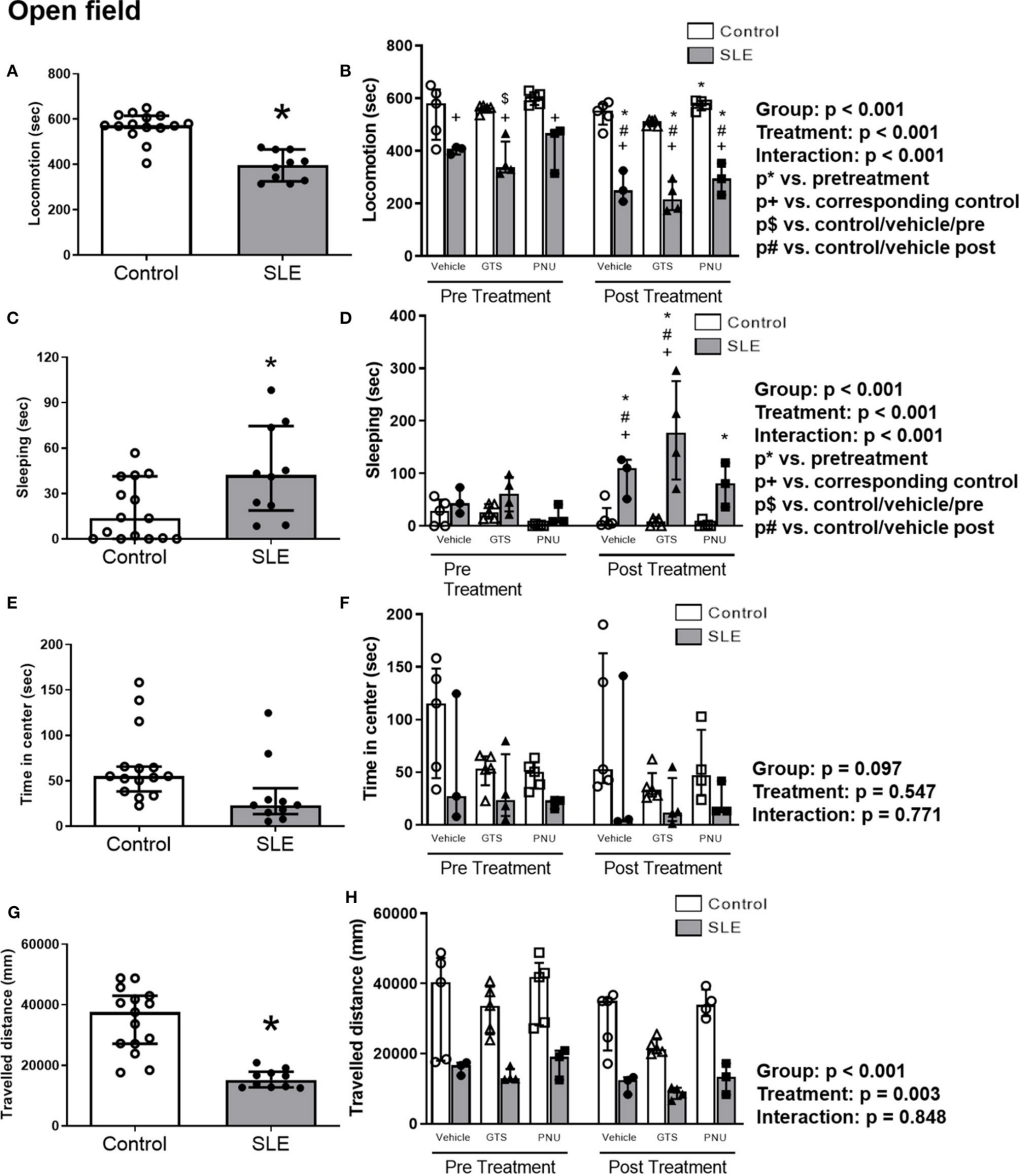
Open field test in control and SLE mice pre-treatment **(A,C,E,G)** and post-treatment with GTS-21 and PNU-120596 **(B,D,F,H)**. The pre-treatment data is a comparison between all control and all SLE mice at baseline. A *t*-test was used to determine significant differences between SLE and control mice pretreatment. SLE mice had lower locomotion **(A)**, spent a larger amount of time sleeping **(C)**, and traveled less during the test than controls **(G)** (all *p* < 0.05). A two-way ANOVA with repeated measures was used to determine differences between all groups post-treatment (**B,D,F,H**; *n* = 3–5 per group). The results of each ANOVA appears on the graph.

To test anxiety like behavior in SLE and possible anxiolytic effects of PAM, animals were submitted to the open field test. SLE mice had less locomotor activity than controls pre-treatment suggesting a baseline anxiety-like behavior in SLE mice (Figure 4A; *p* < 0.001). While SLE mice had even less locomotor activity post-treatment (Figure 4B), PNU and GTS-21 had no significant effect in SLE mice. Since locomotion was decreased further in all SLE mice post-treatment, this suggests that age or disease progression can be responsible for the decrease in locomotor activity in these animals.

SLE mice had more sleeping time during the open field test compared to controls pre-treatment (Figure 4C, *p* = 0.02). Sleeping time increased in SLE mice post-treatment in all treatment groups (vehicle, GTS-21 and PNU) compared to pre-treatment (Figure 4D, *p* < 0.05), but only vehicle- and GTS-21-treated SLE mice had higher sleeping time than corresponding controls post-treatment.

There were no differences observed across groups for time spent in center during the open field test (Figures 4E,F), but distance traveled was decreased in SLE mice compared to controls pre-treatment (Figure 4G, *p* < 0.001). Although there was a significant effect of treatment (*p* = 0.003), there was no interaction (*p* = 0.848) to allow determination of differences within groups in the traveled distance measurements (Figure 4H).

**Figure 5:**
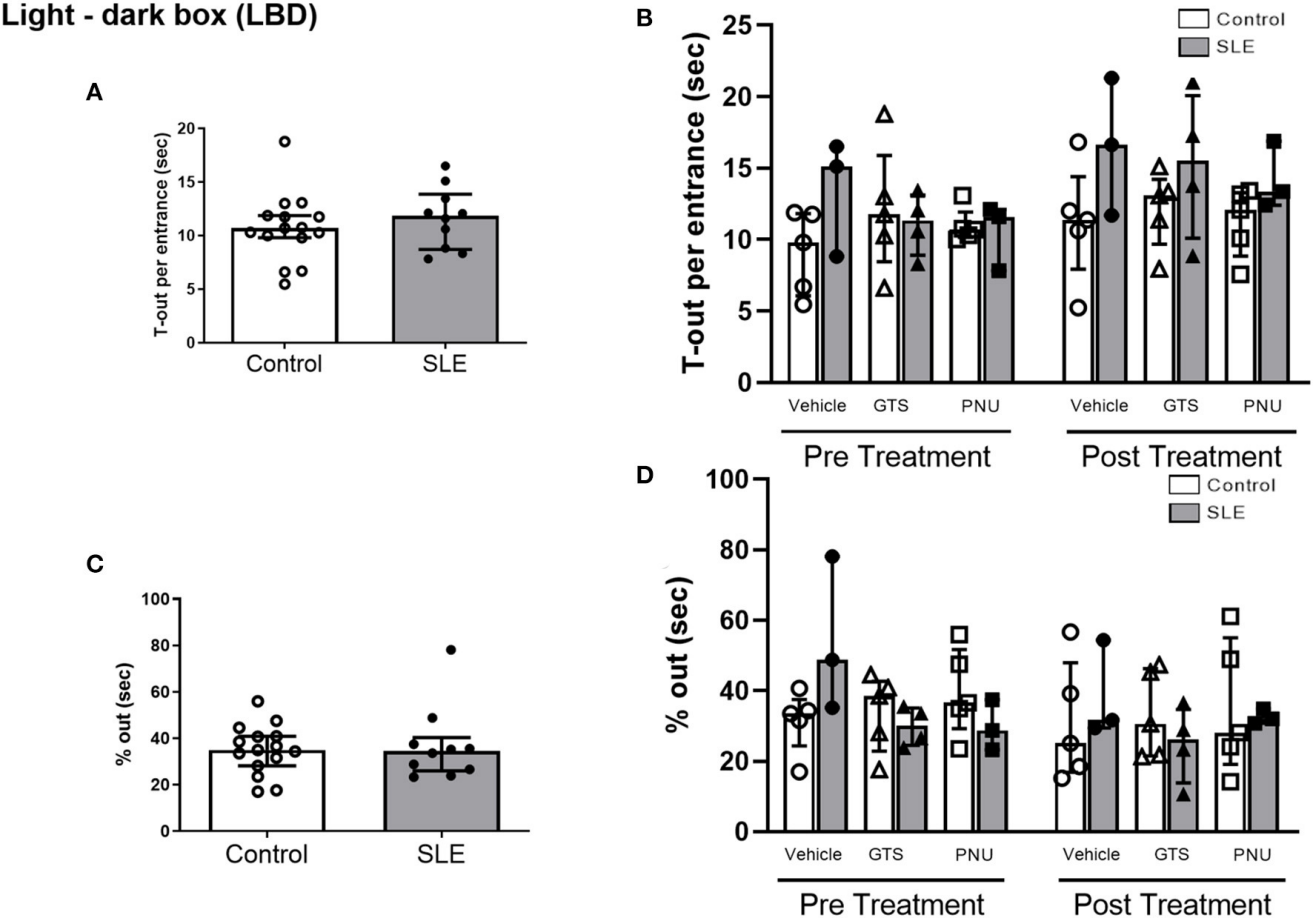
Light-dark box test in control and SLE mice pre-treatment **(A,C)** and post-treatment with GTS-21 and PNU-120596 **(B,D)**. The pre-treatment data is a comparison between all control and all SLE mice at baseline. A *t*-test was used to determine significant differences between SLE and control mice pretreatment and there were no differences **(A,C)**. A two-way ANOVA with repeated measures was used to determine differences between all groups post treatment (**B,D**; *n* = 3–5 per group). The results of each ANOVA appears on the graph.

In order to test unconditioned anxiety response through spontaneous exploratory behaviors, animals underwent to the light-dark box test. There were no significant differences between SLE and controls pre-treatment (Figure 5A). There was also no effect of treatment (*p* = 0.300) and no interaction (*p* = 0.836; Figure 5B) to allow determination of differences within groups in % out quantification post-treatment. Although significant treatment effect (*p* = 0.045) there was no interaction (*p* = 0.777) to allow determination of differences within groups in T-out entrance.

**Figure 6:**
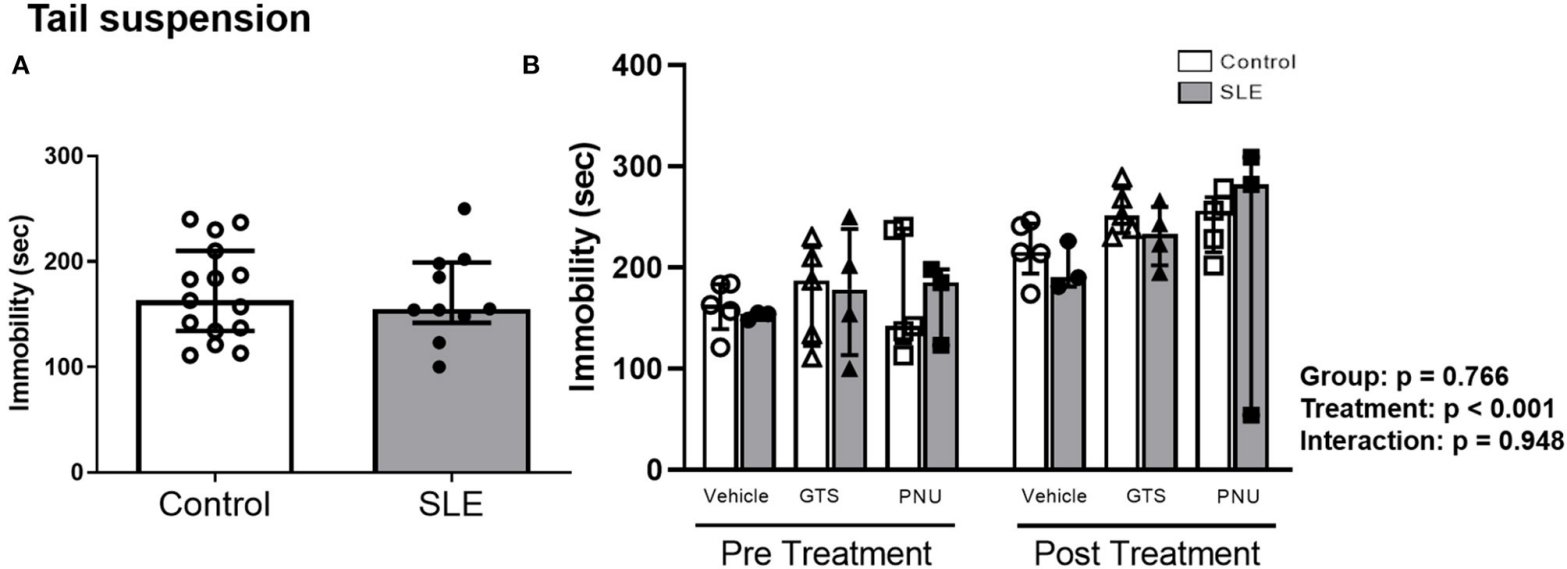
Tail suspension test in control and SLE mice pre-treatment **(A)** and post-treatment with GTS-21 and PNU-120596 **(B)**. The pre-treatment data is a comparison between all control and all SLE mice at baseline. A *t*-test was used to determine significant differences between SLE and control mice pretreatment. There was not a difference in immobility during the suspension test between SLE mice and controls. A two-way ANOVA with repeated measures was used to determine differences between all groups post treatment (**B**; *n* = 3-5 per group). The results of each ANOVA appears on the graph.

To verify the depressive like behavior in SLE mice and the possible effects of PAM, the tail suspension test was performed. There were no differences in the duration of immobility during the tail suspension test between control and SLE animals in pre-treatment (Figure 6A) and although there was an effect of treatment within the groups (*p* < 0.001) there was no interaction (*p* = 0.948) (Figure 6B).

## Discussion

The current study revealed that the use of α7-nAChR modulators in mice with advanced SLE has minimal beneficial effects. The severity of SLE was not altered by either GTS-21 or PNU. In addition, renal inflammaton and renal injury seen in untreated SLE mice with advanced disease were not improved following GTS-21 or PNU administration. The deficits in behavior noted in untreated SLE mice were minimally altered by administration of α7 ligands at this late stage.

The current study was planned as a post-treatment; theoretically this study mimics a late-stage SLE patient with active disease searching for therapy to reverse the vicious sequelae of events resulting from the chronic inflammation. The *NZBWF1* mice used in this study closely mirrors human SLE (48–50). These mice typically start to develop nuclear autantibodies like dsDNA autoantibodies at ~20 weeks of age and these autoantibodies then deposit into tissues like the kidney and initiate inflammatory responses. We have shown in our published data that these inflammatory responses lead to chronic inflammation in the kidney in the form of lupus nephritis and that this inflammation contributes to the hypertension and renal injury frequently seen in these mice by 35 weeks of age (51). Prior studies indicate that systemic administration of nicotine at this late stage was beneficial in reducing renal inflammation and injury (26), but because of the toxic and addictive nature of nicotine, we aimed to determine the efficacy of systemic administration of a less toxic, yet potent alternative—a partial agonists and a selective α7 PAM. We demonstrated in the current study that global GTS-21 and PNU did not have significant effects on splenic and renal inflammation in SLE mice with advance disease, and therefore did not protect from the associated renal injury and hypertension (Figures 3A,B).

In our previously published study we hypothesized that chronic infusion of nicotine would activate the cholinergic anti-inflammatory pathway at the level of splenic α7-nAChRs (26). We know now that the cholinergic anti-inflammatory pathway is indeed impaired in female SLE mice (46, 51). As a PAM, PNU-120596 is inactive in the absence of α7 agonists meaning that a basal cholinergic tone is needed in order to PAM have an effect (29). We have demonstrated that SLE model has a dampened vagal tone (46), so perhaps PNU-120596 was not effective in decreasing inflammation and hypertension due to the lack of cholinergic tone and α7 agonists present in this advanced stage in female SLE mice. Further, there are 9-α (α1-7, α 9-10), 4-β (β1–4), and γ, δ, and ε subunits of the nAChR on the plasma membranes of skeletal muscle cells, neurons, and other non-neuronal cells (52). Therefore, receptors other than α7-nAChR could be involved in the beneficial effects of chronic nicotine and these other receptors may not be altered by GTS-21. Nevertheless, the enthusiam for studying augmented activation of the α7 receptor remains since we only tested the ability of the drugs to reverse advanced SLE. Studies to test the efficacy of α7 ligands at different stages of SLE are still warranted. In addition, a new prototypical metagonist has become available and we hypothesize that this α7 ligand will be more efficacious than previously used α7 ligands since it is highly effective in reducing inflammation and inflammatory pain in multiple rodent models (53). This agent activates primarily non-ionotropic α7-dependent pathways by inducing extremely prolonged states of nearly complete desensitization (i.e., non-conductive states) of α7 nAChRs (54). Thus, our future studies will test a novel concept and hypothesis that robust α7 desensitization and activation of α7-nAChR non-conductive states will effectively inhibit chronic inflammation associated with physiological and behavioral deficits in a mouse model of SLE.

In a set of behavioral assays, we explored the therapeutic utility of sub-chronic GTS-21 and PNU exposure. The drug-like properties of GTS-21 (EC_50_~2.9 μM) (55) and PNU (EC_50_~1.5 μM) (56) are advantageous for central nervous system therapeutics, including rapid brain penetration and efficacy. PNU-120596 (<30 mg/kg) was found to be safe in rodents (57, 58), but GTS-21 was found to be safe in humans as major side effects have not been detected after oral administration of large doses of GTS-21 (e.g., <450 mg/day) (59, 60). GTS-21 is rapidly metabolized to its 4-hydroxy metabolite, 4OH-GTS-21. In a direct comparison study of 4OH-GTS-21 vs. GTS-21 (55): 4OH-GTS-21 was found to be 30–50% more efficacious and potent than GTS-21 for both rat [EC_50_(4OH-GTS-21)~1.6 μM vs. EC_50_(GTS-21)~2.9 μM] and human [EC_50_(4OH-GTS-21)~4.0 μM vs. EC_50_(GTS-21)~6.0 μM] α7-nAChRs. 4OH-GTS-21 was found to be less efficacious to cause residual inhibition than GTS-21 for both rat and human α7-nAChRs with IC_50_ = 356 μM (rat) and >1 mM (human) as compared to IC_50_ = 25 μM (rat) and 82 μM (human) for GTS-21 (55). These electrophysiological assays revealed 4OH-GTS-21 as a superior α7 agonist as compared to GTS-21 and suggested that therapeutic effects of GTS-21 may largely reflect its pro-drug activity rather than direct agonism.

While exogenous orthostatic α7-nAChR agonists like GTS-21 present valuable pharmacological tools, therapeutic approaches that could amplify the brain's innate anti-inflammatory efficacy present potentially powerful therapeutic alternatives and have not been fully explored (29). α7 PAM only amplify α7-dependent endogenous cholinergic tone in a spatially and temporally restricted manner (29), creating a potential for differential efficacy and an improved safety profile as compared to α7 agonists that activate α7-nAChRs indiscriminately. Thus, α7 PAM-based treatments in chronic inflammatory disorders like SLE may enhance endogenous auto-therapy by converting endogenous acetylcholine into potent therapeutic agent. Because α7 PAM are inactive in the absence of α7 agonists, α7 PAM can be accurately delivered to the site of inflammation by a routine systemic administration: α7 PAM will be homogeneously distributed throughout the body by circulation, but their therapeutic action will occur only near the site and time of elevated levels of endogenous acetylcholine (e.g., vagus nerve stimulation) (29). Therefore, α7-PAM introduce a novel therapeutic concept that is based on a substantively different mechanism: inflammation-induced recruitment and potentiation of endogenous α7-dependent cholinergic anti-inflammatory pathway (29, 61).

Evidence in the literature shows a high prevalence of behavioral disorders in SLE patients as well as SLE mice (62–64). *NZBWF1* mice manifest anxiety-like phenotypes and decreased locomotor activity. This phenotype is exacerbated by interferon-α suggesting that inflammation has an important role on deficits in behavior in this model (65). In the present study, behavioral assays detected significant differences between control and SLE groups (Figures 4–6), but both GTS-21 and PNU failed to generate therapeutic benefits in either group. This suggests that at the time of treatment (weeks 33–35) sensorimotor and emotional deficits may have matured to the point of no return. In the context of obtained data it would be informative to determine the effects of chronic exposure to GTS-21 and PNU at earlier time points (e.g., weeks 10–30) to test the hypothesis that α7 therapies may impede the disease development.

Although we found the GTS-21 and PNU did not contribute significantly in reducing inflammation and improving behavior, there were some limitations to our current study and notable findings that suggest that treating SLE mice at an earlier stage with potent α7 ligands could be beneficial. First, the end of the study coincides with the average lifespan of female SLE mice (i.e, *NZBWF1* mice) used in this study −35 weeks—and it is known that SLE pathology and morbidity worsens with age. It is likely that SLE mice at this advanced stage have reached a point of irreversibility in renal inflammation and renal injury and this explains why we saw no effect of α7 ligands. Future studies that use an earlier timeline for administration of α7 ligands, e.g., at 20 weeks of age when plasma dsDNA autoantibodies start to develop, could inform us if this novel therapy is efficacious in preventing the development and maintenance of chronic inflammation and therefore protecting from end stage renal disease and hypertension among other morbidities in SLE. Lastly, we measured renal and splenic TNF in this study, a common inflammatory mediator involved in renal disease and hypertension. A more involved temporal analysis of renal immune cells and inflammatory mediators would aid in determining if modulating the α7-nAChR is indeed a viable therapeutic option in SLE.

The studies discussed here within pertaining to manipulation of the cholinergic anti-inflammatory pathway are highly clinically relevant. Manipulation of the components of this pathway via pharmacological means should prove to be efficacious in human SLE as in mouse models. The current study focused on pharmacological activation of the downstream α7nAChR, but stimulation of the efferent vagus nerve is the main trigger that turns on this anti-inflammatory pathway and can be targeted pharmacologically. We have shown that pharmacological potentiation of the efferent vagus nerve attenuates blood pressure in female SLE mice (46), but other studies involving vagus nerve stimulation have already been explored in humans. A recently completed clinical trial exploring transcutaneous auricular vagus nerve stimulation demonstrated its effectiveness in reducing pain and fatigue in patients with SLE (66). Together, these findings are confirmation of the importance of the vagally-mediated cholinergic anti-inflammatory pathway in SLE and they highlight the need for understanding the mechanisms involved.

## Data Availability Statement

The raw data supporting the conclusions of this article will be made available by the authors, without undue reservation.

## Ethics Statement

The animal study was reviewed and approved by University of North Texas Health Science Center's Institutional Animal Care and Use Committee (IACUC).

## Author Contributions

VU and KM conceived and designed study. JM, CY-S, VU, and KM performed experiments. JM, CY-S, CS, VU, and KM analyzed data. JM, CY-S, CS, VU, and KM interpreted results. JM, CY-S, CS, VU, and KM prepared figures. VU and KM drafted manuscript. JM, CY-S, CS, WK, VU, and KM edited and revised manuscript. JM, CY-S, CS, WK, VU, and KM approved final version of manuscript. All authors contributed to the article and approved the submitted version.

## Conflict of Interest

The authors declare that the research was conducted in the absence of any commercial or financial relationships that could be construed as a potential conflict of interest.
